# Safety of COVID-19 Vaccines Among Pregnant Women in India: A Systematic Review and Meta-Analysis

**DOI:** 10.7759/cureus.86115

**Published:** 2025-06-16

**Authors:** Aravind P Gandhi, Priyanka Yadav, Anuva Kapoor, Nishaant Ramasamy, Mogan Kaviprawin, Kavinkumar Saravanan, Anita Yadav, Abhijit Choudhary, Felista K Joseph

**Affiliations:** 1 Department of Community Medicine, All India Institute of Medical Sciences, Nagpur, Nagpur, IND; 2 Department of Community Medicine, South Asia Field Epidemiology and Technology Network, Inc., Chennai, IND; 3 Department of Community Medicine, Swamy Vivekanandha Medical College Hospital and Research Institute, Namakkal, IND; 4 Department of Obstetrics and Gynecology, All India Institute of Medical Sciences, Nagpur, Nagpur, IND; 5 Department of Pediatrics, All India Institute of Medical Sciences, Nagpur, Nagpur, IND; 6 Department of Pathology, Melmaruvathur Adhiparasakthi Institute of Medical Sciences and Research, Melmaruvathur, IND

**Keywords:** adverse events following immunization, covid-19 vaccine, fetomaternal outcomes, india, pregnancy

## Abstract

Coronavirus disease 2019 (COVID-19) documented an estimated decrease in life expectancy of 1.6 years globally. Pregnant women exhibited higher infection rates and experienced prolonged illness durations, often exceeding four months. COVID-19 was also related to adverse maternal morbidity and near-miss incidents. In India, although there are studies on the effectiveness of COVID-19 vaccines among the overall population, the effectiveness in preventing infections among pregnant women is not well documented. The present meta-analysis pooled the safety of the COVID-19 vaccine among pregnant women in India. The search was conducted among the major electronic databases: PubMed, Scopus, Web of Science, and Cochrane (date of search: November 30, 2024). Screening, risk of bias (ROB), and data extraction of the studies were undertaken by two independent reviewers, with adjudication of conflicts by a third reviewer. We described the pooled effectiveness of vaccination on adverse events following immunization (AEFI) and fetomaternal outcomes using RStudio (Posit Software, Boston, MA). The I² statistic assessed the heterogeneity among studies. We conducted a Grading of Recommendations Assessment, Development, and Evaluation (GRADE) assessment to ascertain the certainty of the results. Database search yielded 215 unique articles. We included nine eligible studies for the analysis, including 5,630 pregnant women. Covishield and Covaxin were the vaccines taken by them. The pooled odds ratio (OR) for preterm labor in mothers with COVID-19 vaccination was 0.62 (95% confidence interval (CI): 0.49-0.79), with low certainty. The OR for low birth weight was 0.88 (95% CI: 0.06-13.71). AEFI rates were similar between the vaccinated pregnant and non-pregnant women (OR: 0.88, 95% CI: 0.00-268.11). No studies on efficacy and effectiveness could be found. Our pooled analysis concluded that COVID-19 vaccination among pregnant women in India is not associated with the increased risk of AEFIs or maternal or fetal outcomes.

## Introduction and background

Coronavirus disease 2019 (COVID-19) led to an estimated decrease in life expectancy of 1.6 years globally, with India showing a decline of 1.8 years between 2019 and 2021 [1]. In 2021, COVID-19 was reported as a common reason for mortality and disability-adjusted life years, surpassing ischemic heart diseases [2]. India reported over 45 million infections and five lakh deaths due to COVID-19 [3]. Low- and middle-income countries, including India, reported higher COVID-19 infection rates (0.88%), and around one in 10 infected individuals was hospitalized [4]. Pregnant women, being a distinct vulnerable population, had a higher incidence of infection with a prolonged illness lasting over four months, impacting a major part of the antenatal period [5]. Physiological changes during pregnancy modulate the maternal immune system, suppressing immunity, especially in the later trimesters, leading to increased referral to critical care units with ventilatory support [6]. COVID-19 was also related to adverse maternal morbidity and near-miss incidents. These concerns have underscored the urgency of implementing protective measures, including vaccination, in this population.

In India, two COVID-19 vaccines, i.e., Covaxin (inactivated SARS-CoV-2) and Covishield (adenovirus vector with SARS-CoV-2 spike protein), with demonstrated efficacy, were rolled out in 2021. Initially, individuals aged ≥60 years and those aged over 45 years with comorbidities were vaccinated; later, the population between 18 and 45 years was enrolled. Covishield showed an effectiveness of around 60% in multiple countries, including India, which remained consistent with the emergence of the delta wave [7,8]. Similarly, Covaxin had an overall effectiveness of 71% in the Indian setting [9]. Although there are studies on the effectiveness of COVID-19 vaccines among the overall population, the effectiveness in preventing infections among pregnant women is not well documented. In addition, pregnant women were excluded from initial clinical trials due to ethical and safety concerns, leaving a critical knowledge gap regarding vaccine safety, efficacy, and effectiveness in this high-risk group [10].

Around 30 million pregnancies are reported per year in India. India is one of the Southeast Asian countries with high maternal and newborn mortality [11]. India had 6.5% of pregnant women with severe maternal outcomes and 5.1% near-miss incidence during the COVID-19 pandemic, which is significantly higher than in the pre-COVID-19 era [11]. During mid-2021, following the emergence of evidence on vaccine efficacy among pregnant women, the Health Ministry of India revised the implementation guidelines, allowing pregnant women to choose vaccination after informed consent [12]. Both Covaxin and Covishield were introduced to pregnant women in 2021. Studies showed that the antibodies that emerged in pregnant women offer passive immunity through transplacental and breast milk routes [13]. Despite these promising findings, concerns about vaccine safety, adverse events, and potential impacts on pregnancy outcomes persisted, especially in low-resource settings. This could significantly impact the acceptance and uptake of the vaccination among pregnant women in countries like India. In addition, the profile of effectiveness by type of COVID-19 vaccine in India is not reported. This meta-analysis intended to pool the safety, efficacy, and effectiveness of the COVID-19 vaccine among pregnant women in India.

## Review

Methods

The current systematic review and meta-analysis (SRMA) adapted the Preferred Reporting Items for Systematic Reviews and Meta-Analyses (PRISMA) 2020 guidelines (Appendices).

Eligibility Criteria

The research question addressed in the SRMA was framed into a PECO format to identify the eligible population, exposure, comparator group, and outcomes documented (Table 1).

**Table 1:** Inclusion and exclusion criteria for the systematic review and meta-analysis COVID-19: coronavirus disease 2019, AEFI: adverse events following immunization, RCT: randomized controlled trial

Eligibility criteria	Inclusion	Exclusion
Participants	Pregnant women: any trimester, any age, with or without pre-existing comorbidities	-
Exposure	COVID-19 vaccine: any type of vaccine, any dose, any number of doses	-
Comparator	Placebo, no vaccine, no comparator group (single-group studies with vaccinated group only)	-
Outcomes	Primary outcomes: AEFIs, mortality, fetomaternal outcomes, pregnancy-related complications, antibody titer, breakthrough infections	-
Study designs	Cross-sectional, prevalence studies, case-control, cohort studies, interventional studies (RCTs, non-RCTs, and quasi-experimental studies)	Qualitative, policy, case reports, and opinion reports
	Databases: MEDLINE (PubMed), Scopus, Embase, Web of Science, Cochrane CENTRAL; other sources: citation searching of the eligible studies; geography: India; date of search: published until November 30, 2024; English-language human studies	-
	Published and unpublished data	-

We included studies conducted among pregnant women of any age with or without pre-existing comorbidities in India. Pregnant women vaccinated against COVID-19 during any trimester were included. Exposure is defined as the receipt of the COVID-19 vaccine and/or the number of doses given to the study population. Studies with any of the following comparator groups were included in the SRMA: pregnant women who were not vaccinated against COVID-19 (or given placebo) to estimate the effect on fetomaternal outcomes and non-pregnant women who had taken COVID-19 vaccines to analyze the safety in the context of adverse events following immunization (AEFI) in pregnancy. Studies with a single arm that assessed outcomes among vaccinated pregnant women were also included. Primary outcomes include AEFIs, maternal mortality, fetomaternal outcomes, pregnancy-related complications, antibody titers post-vaccination, and breakthrough infections. Appropriate effect measures were abstracted from the literature.

Search Strategy

The SRMA included published literature observing vaccine efficacy or effectiveness, including pregnant women reported from India. A preliminary search strategy was built by one of the authors, which was peer-reviewed by two other authors. Following the peer review, the search strategy was modified and finalized. A final search strategy was implemented among the major electronic databases: PubMed, Scopus, Web of Science, and Cochrane. All studies published until November 30, 2024, in the above databases were included. Boolean operators and combinations of key terms were utilized, and the detailed search strategy has been enumerated in Table 2. Only English-language articles involving human participants were included. The reference lists of the eligible studies were also screened manually to ensure completeness.

**Table 2:** Adjusted search terms as per searched electronic databases (as of November 30, 2024)

Database	Number	Search query	Results
PubMed	#1	((((((Pregnant Women[MeSH Terms]) OR (Pregnancy[MeSH Terms])) OR (Pregnan*[Title/Abstract])) OR (gestation[Title/Abstract])) ) OR (Antenatal[Title/Abstract])) OR (mother*[Title/Abstract])	1,381,014
	#2	("covid 19 vaccines"[MeSH Terms] OR "covid 19 vaccine*"[Title/Abstract] OR "covid19 vaccine*"[Title/Abstract] OR "coronavirus disease 2019 vaccine*"[Title/Abstract] OR "sars cov2 vaccine*"[Title/Abstract] OR ("BNT162"[All Fields] AND "vaccines"[MeSH Terms]) OR ("mRNA-1273"[All Fields] AND "vaccines"[MeSH Terms]) OR (("ad26 cov2 s vaccine"[All Fields] OR "Ad26.COV2.S"[All Fields]) AND "vaccines"[MeSH Terms]) OR ("ChAdOx1"[All Fields] AND "covid 19 vaccines"[MeSH Terms]) OR ("Ad5-nCoV"[All Fields] AND "vaccines"[MeSH Terms]) OR (("SARS-CoV-2"[MeSH Terms] OR "SARS-CoV-2"[All Fields] OR "sars2"[All Fields]) AND "vaccine*"[Title/Abstract]) OR "sars cov 2 vaccine*"[Title/Abstract] OR "2019 ncov vaccine*"[Title/Abstract] OR "2019 ncov vaccine*"[Title/Abstract] OR "covid 19 vaccine*"[Title/Abstract] OR "sars coronavirus 2 vaccine*"[Title/Abstract] OR "bnt162 vaccine"[Title/Abstract] OR "pfizer biontech covid 19 vaccine"[Title/Abstract] OR "BNT162b1"[Title/Abstract] OR "BNT162b2"[Title/Abstract] OR "Comirnaty"[Title/Abstract] OR "mrna 1273 vaccine"[Title/Abstract] OR "covid 19 vaccine mrna 1273"[Title/Abstract] OR (("SARS-CoV-2"[MeSH Terms] OR "SARS-CoV-2"[All Fields] OR "2019-nCoV"[All Fields]) AND "vaccine mrna 1273"[Title/Abstract]) OR ("mRNA-1273"[All Fields] AND "2019 ncov vaccine"[Title/Abstract]) OR "mrna 1273 covid 19 vaccine"[Title/Abstract] OR "mrna 1273 vaccine"[Title/Abstract] OR "moderna covid 19 vaccine"[Title/Abstract] OR "ad26 cov2 s vaccine"[Title/Abstract] OR "Ad26.COV2.S"[Title/Abstract] OR (("ad26 cov2 s vaccine"[All Fields]) AND "covid 19 vaccine"[Title/Abstract]) OR "JNJ-78436735"[Title/Abstract] OR "JNJ-78436735"[Title/Abstract] OR "chadox1 covid 19 vaccine"[Title/Abstract] OR (("ChAdOx1"[All Fields] AND ("SARS-CoV-2"[MeSH Terms] OR "SARS-CoV-2"[All Fields] OR "sars2"[All Fields])) AND "Vaccine"[Title/Abstract]) OR "chadox1 ncov 19"[Title/Abstract] OR "Covishield"[Title/Abstract] OR "AZD1222"[Title/Abstract] OR "oxford astrazeneca covid 19 vaccine"[Title/Abstract] OR "Vaxzevria"[Title/Abstract] OR "COVAXIN"[Title/Abstract] OR "sputnik v"[Title/Abstract] OR ("Sputnik"[All Fields] AND "Light"[Title/Abstract]) OR "Gam-COVID-Vac"[Title/Abstract] OR "Sinovac"[Title/Abstract] OR "CoronaVac"[Title/Abstract] OR "Sinopharm"[Title/Abstract] OR "covid 19 vaccine janssen"[Title/Abstract] OR "BBIBP-CorV"[Title/Abstract] OR "EpiVacCorona"[Title/Abstract] OR (("covid 19 vaccines"[MeSH Terms] OR ("covid 19"[All Fields] AND "vaccines"[All Fields]) OR "covid 19 vaccines"[All Fields] OR "covid 19 vaccine"[All Fields]) AND "Ad5-nCoV"[Title/Abstract]) OR ((("f8 protein human"[Supplementary Concept] OR "f8 protein human"[All Fields] OR "recombinate"[All Fields] OR "recombinant"[All Fields] OR "recombinants"[All Fields] OR "recombinated"[All Fields] OR "recombinates"[All Fields] OR "recombination, genetic"[MeSH Terms] OR ("recombination"[All Fields] AND "genetic"[All Fields]) OR "genetic recombination"[All Fields] OR "recombination"[All Fields] OR "recombinations"[All Fields] OR "recombinational"[All Fields] OR "recombinative"[All Fields] OR "recombine"[All Fields] OR "recombined"[All Fields] OR "recombineered"[All Fields] OR "recombineering"[All Fields] OR "recombines"[All Fields] OR "recombining"[All Fields]) AND ("SARS-CoV-2"[MeSH Terms] OR "SARS-CoV-2"[All Fields] OR ("novel"[All Fields] AND "Coronavirus"[All Fields]) OR "novel coronavirus"[All Fields]) AND ("vaccin"[Supplementary Concept] OR "vaccin"[All Fields] OR "vaccination"[MeSH Terms] OR "vaccination"[All Fields] OR "vaccinable"[All Fields] OR "vaccinal"[All Fields] OR "vaccinate"[All Fields] OR "vaccinated"[All Fields] OR "vaccinates"[All Fields] OR "vaccinating"[All Fields] OR "vaccinations"[All Fields] OR "vaccination s"[All Fields] OR "vaccinator"[All Fields] OR "vaccinators"[All Fields] OR "vaccine s"[All Fields] OR "vaccined"[All Fields] OR "vaccines"[MeSH Terms] OR "vaccines"[All Fields] OR "Vaccine"[All Fields] OR "vaccins"[All Fields])) AND "Ad5-nCoV"[Title/Abstract]) OR ("Ad5-nCoV"[All Fields] AND "covid 19 vaccine"[Title/Abstract]) OR ((("f8 protein human"[Supplementary Concept] OR "f8 protein human"[All Fields] OR "recombinate"[All Fields] OR "recombinant"[All Fields] OR "recombinants"[All Fields] OR "recombinated"[All Fields] OR "recombinates"[All Fields] OR "recombination, genetic"[MeSH Terms] OR ("recombination"[All Fields] AND "genetic"[All Fields]) OR "genetic recombination"[All Fields] OR "recombination"[All Fields] OR "recombinations"[All Fields] OR "recombinational"[All Fields] OR "recombinative"[All Fields] OR "recombine"[All Fields] OR "recombined"[All Fields] OR "recombineered"[All Fields] OR "recombineering"[All Fields] OR "recombines"[All Fields] OR "recombining"[All Fields]) AND ("SARS-CoV-2"[MeSH Terms] OR "SARS-CoV-2"[All Fields] OR ("novel"[All Fields] AND "Coronavirus"[All Fields]) OR "novel coronavirus"[All Fields]) AND ("vaccin"[Supplementary Concept] OR "vaccin"[All Fields] OR "vaccination"[MeSH Terms] OR "vaccination"[All Fields] OR "vaccinable"[All Fields] OR "vaccinal"[All Fields] OR "vaccinate"[All Fields] OR "vaccinated"[All Fields] OR "vaccinates"[All Fields] OR "vaccinating"[All Fields] OR "vaccinations"[All Fields] OR "vaccination s"[All Fields] OR "vaccinator"[All Fields] OR "vaccinators"[All Fields] OR "vaccine s"[All Fields] OR "vaccined"[All Fields] OR "vaccines"[MeSH Terms] OR "vaccines"[All Fields] OR "Vaccine"[All Fields] OR "vaccins"[All Fields]) AND ("adenovirally"[All Fields] OR "adenoviridae"[MeSH Terms] OR "adenoviridae"[All Fields] OR "adenoviral"[All Fields])) AND "vector5"[Title/Abstract]) OR ("nCoV-19"[Title/Abstract]) OR (("covid 19 vaccines"[MeSH Terms] OR ("covid 19"[All Fields] AND "vaccines"[All Fields]) OR "covid 19 vaccines"[All Fields] OR "2019 ncov vaccine"[All Fields]) AND "Ad5-nCoV"[Title/Abstract]) OR ("Ad5-nCoV"[All Fields] AND "2019 ncov vaccine"[Title/Abstract]) OR "BBV152"[Title/Abstract])	54,930
	#3	(((((((((((((safety[MeSH Terms]) OR (Vaccine Efficacy[MeSH Terms])) OR (Mortality[MeSH Terms])) OR (hospitalization[MeSH Terms])) OR (safety[Title/Abstract])) OR (adverse event*[Title/Abstract])) OR (AEFI[Title/Abstract])) OR (hospitalization*[Title/Abstract])) OR (mortality[Title/Abstract])) OR (death*[Title/Abstract])) OR (efficacy[Title/Abstract])) OR (effectiveness[Title/Abstract])) OR ((((feto-maternal outcome*[Title/Abstract]) OR (fetomaternal outcome*[Title/Abstract])) OR (pregnancy outcome*[Title/Abstract])) OR (pregnancy outcome[MeSH Terms]))) OR ((Adverse neonatal outcome*[Title/Abstract]) OR (((((((((((perinatal death[Title/Abstract]) OR (preterm birth[Title/Abstract])) OR (small for gestational age[Title/Abstract])) OR (low birth weight[Title/Abstract])) OR (Apgar score[Title/Abstract])) OR (neonatal hypoglycemia[Title/Abstract])) OR (neonatal jaundice[Title/Abstract])) OR (neonatal respiratory distress[Title/Abstract])) OR (neonatal infection*[Title/Abstract])) OR (congenital malformation*[Title/Abstract])) OR ((((((((Neonatal Respiratory Distress Syndrome[MeSH Terms]) OR (Congenital Abnormality[MeSH Terms])) OR (perinatal death[MeSH Terms])) OR (preterm birth[MeSH Terms])) OR (low birth weight[MeSH Terms])) OR (neonatal hypoglycemia[MeSH Terms])) OR (neonatal jaundice[MeSH Terms])))) OR ((((((Adverse Birth Outcomes[MeSH Terms]) OR (Adverse Birth Outcome*[Title/Abstract])) OR (Adverse pregnancy outcome*[Title/Abstract])) OR (Adverse delivery outcome*[Title/Abstract])) OR ((((((((((gestational diabetes[Title/Abstract]) OR (preeclampsia[Title/Abstract])) OR (maternal infection*[Title/Abstract])) OR (antepartum hemorrhage[Title/Abstract])) OR (placental abruption[Title/Abstract])) OR (premature rupture of membranes[Title/Abstract])) OR (PROM[Title/Abstract])) OR (induction of labour[Title/Abstract])) OR (mode of delivery[Title/Abstract])) OR (postpartum hemorrhage[Title/Abstract]))) OR ((((((gestational diabetes[MeSH Terms]) OR (preeclampsia[MeSH Terms])) OR (placental abruption[MeSH Terms])) OR (premature rupture of membranes[MeSH Terms])) OR (induction of labour[MeSH Terms])) OR (postpartum hemorrhage[MeSH Terms]))))	5,623,184
	#4	"India"[MeSH Terms] OR "india*"[Title/Abstract] OR "India"[Affiliation]	895,766
	#5	#1 AND #2 AND #3 AND #4	73
Embase	#1	pregnant AND ('women'/exp OR 'women'/exp) OR 'pregnancy'/exp OR 'gestation'/exp OR gestation*:ab,kw,ti OR antenatal:ab,kw,ti OR 'mother'/exp OR mother*:ab,ti,kw	1,638,082
	#2	('sars-cov-2 vaccine' OR covid19) AND vaccine*	74,857
	#3	'safety'/exp OR safety:ab,kw,ti OR 'vaccine effectiveness'/exp OR effiacy:ab,kw,ti OR effectiveness:ab,kw,ti OR 'fetomaternal outcome'/exp OR 'fetomaternal outcome':ab,kw,ti OR 'adverse event'/exp OR 'adverse event*':ab,kw,ti OR 'hospitalization'/exp OR 'hospitalization':ab,kw,ti OR 'mortality'/exp OR 'mortality':ab,kw,ti OR 'death':ab,kw,ti OR 'pregnancy outcome'/exp OR 'pregnancy outcome*' OR 'aefi*':ab,kw,ti	6,626,591
	#4	'adverse neonatal outcome'/exp OR 'adverse neonatal outcome' OR 'perinatal death'/exp OR 'perinatal death' OR 'prematurity'/exp OR 'prematurity' OR 'preterm birth':ab,kw,ti OR 'small for gestational age'/exp OR 'small for gestational age' OR 'low birth weight'/exp OR 'low birth weight' OR 'apgar score'/exp OR 'apgar score' OR 'neonatal hypoglycemia'/exp OR 'neonatal hypoglycemia' OR 'newborn jaundice'/exp OR 'newborn jaundice' OR 'neonatal jaundice':ab,kw,ti OR 'neonatal respiratory distress syndrome'/exp OR 'neonatal respiratory distress syndrome' OR 'neonatal respiratory distress':ab,kw,ti OR 'newborn infection'/exp OR 'newborn infection' OR 'neonatal infection':ab,kw,ti OR 'congenital malformation'/exp OR 'congenital malformation' OR 'congenital disorder'/exp OR 'congenital disorder' OR 'congenital abnormality':ab,kw,ti OR 'adverse birth outcome':ab,kw,ti	2,283,693
	#5	'adverse pregnancy outcome'/exp OR 'adverse pregnancy outcome' OR 'adverse delivery outcome*':ab,kw,ti OR 'gestational diabetes'/exp OR 'gestational diabetes' OR 'preeclampsia'/exp OR 'preeclampsia' OR 'maternal infection'/exp OR 'maternal infection' OR 'antepartum hemorrhage'/exp OR 'antepartum hemorrhage' OR 'solutio placentae'/exp OR 'solutio placentae' OR 'placental abruption':ab,kw,ti OR 'premature rupture of membranes'/exp OR 'premature rupture of membranes' OR prom:ab,kw,ti OR 'induction of labour':ab,kw,ti OR 'mode of delivery'/exp OR 'mode of delivery' OR 'postpartum hemorrhage'/exp OR 'postpartum hemorrhage'	217,088
	#6	#3 OR #4 OR #5	8,580,104
	#7	'india'/exp OR 'india':ab,kw,ti,ff,ad	1,316,451
	#8	#1 AND #2 AND #6 AND #7	155
Web of Science	#1	(((TS=(pregnan*)) OR TS=(gestation)) OR TS=(antenatal)) OR TS=(mother*)	848,327
	#2	(((((((((((((TS=("sars-cov-2 vaccine")) OR TS=("COVID-19 vaccine*")) OR TS=("COVID19 vaccine*")) OR TS=(COVISHIELD)) OR TS=(COVAXIN)) OR TS=(astrazeneca )) OR TS=(sputnik)) OR TS=("COVID-Vac")) OR TS=("Sinopharm")) OR TS=(janssen)) OR TS=(moderna)) OR TS=(Vaxzevria)) OR TS=(BNT162b1)) OR TS=("Pfizer-BioNTech")	25,917
	#3	((((((((((TS=(Safety)) OR TS=(efficacy)) OR TS=(mortality)) OR TS=(hospitalization)) OR TS=("adverse event*")) OR TS=(AEFI)) OR TS=(death*)) OR TS=(effectiveness)) OR TS=("feto maternal outcome*")) OR TS=("fetomaternal outcome*")) OR TS=("pregnancy outcome*")	5,230,414
	#4	TS=((Adverse neonatal outcome OR perinatal death OR preterm birth OR small for gestational age OR low birth weight OR Apgar score OR neonatal hypoglycemia OR neonatal jaundice OR neonatal respiratory distress OR neonatal infection OR congenital malformation OR Neonatal Respiratory Distress Syndrome OR Congenital Abnormality OR Adverse Birth Outcomes))	247,767
	#5	TS=((Adverse pregnancy outcome* OR Adverse delivery outcome* OR gestational diabetes OR preeclampsia OR maternal infection OR antepartum hemorrhage OR placental abruption OR premature rupture of membranes OR PROM OR induction of labour OR mode of delivery OR postpartum hemorrhage) )	180,048
	#6	#3 OR #4 OR #5	5,493,050
	#7	TS=(India)	238,549
	#8	#1 AND #2 AND #6 AND #7	11
	Scopus	#1	( TITLE-ABS-KEY ( pregnan* ) OR TITLE-ABS-KEY ( mother* ) OR TITLE-ABS-KEY ( antenatal ) OR TITLE-ABS-KEY ( gestation* ) )	1,804,766
#2		( TITLE-ABS-KEY ( covid 19 vaccine* ) OR TITLE-ABS-KEY ( sars AND cov2 AND vaccine* ) OR TITLE-ABS-KEY ( pfizer AND biontech ) OR TITLE-ABS-KEY ( moderna ) OR TITLE-ABS-KEY ( vaxzevria ) OR TITLE-ABS-KEY ( astrazeneca ) OR TITLE-ABS-KEY ( sputnik ) OR TITLE-ABS-KEY ( sinovac ) OR TITLE-ABS-KEY ( covishield ) OR TITLE-ABS-KEY ( covaxin ) )	83,271
#3		( TITLE-ABS-KEY ( safety ) OR TITLE-ABS-KEY ( efficacy ) OR TITLE-ABS-KEY ( mortality ) OR TITLE-ABS-KEY ( hospitalization ) OR TITLE-ABS-KEY ( adverse AND event* ) OR TITLE-ABS-KEY ( aefi ) OR TITLE-ABS-KEY ( aefi AND adverse AND events AND following AND immunization ) OR TITLE-ABS-KEY ( effectiveness ) OR TITLE-ABS-KEY ( feto AND maternal AND outcome* ) OR TITLE-ABS-KEY ( pregnancy AND outcome* ) OR TITLE-ABS-KEY ( "Adverse neonatal outcome*" ) OR TITLE-ABS-KEY ( "perinatal death" ) OR TITLE-ABS-KEY ( "preterm birth" ) OR TITLE-ABS-KEY ( "small for gestational age" ) OR TITLE-ABS-KEY ( "low birth weight" ) OR TITLE-ABS-KEY ( "Apgar score" ) OR TITLE-ABS-KEY ( "neonatal hypoglycemia" ) OR TITLE-ABS-KEY ( "neonatal jaundice" ) OR TITLE-ABS-KEY ( "neonatal respiratory distress" ) OR TITLE-ABS-KEY ( "neonatal infection*" ) OR TITLE-ABS-KEY ( "congenital malformation*" ) OR TITLE-ABS-KEY ( "Neonatal Respiratory Distress Syndrome" ) OR TITLE-ABS-KEY ( "Congenital Abnormalit*" ) OR TITLE-ABS-KEY ( "perinatal death" ) OR TITLE-ABS-KEY ( "delivery outcome*" ) OR TITLE-ABS-KEY ( "Birth Outcome*" ) OR TITLE-ABS-KEY ( "gestational diabetes" ) OR TITLE-ABS-KEY ( preeclampsia ) OR TITLE-ABS-KEY ( "maternal infection*" ) OR TITLE-ABS-KEY ( "antepartum hemorrhage" ) OR TITLE-ABS-KEY ( "placental abruption" ) OR TITLE-ABS-KEY ( "premature rupture of membranes" ) OR TITLE-ABS-KEY ( prom ) OR TITLE-ABS-KEY ( "induction of labour" ) OR TITLE-ABS-KEY ( "mode of delivery" ) OR TITLE-ABS-KEY ( "postpartum hemorrhage" ) )	8,965,013
#4		( TITLE-ABS-KEY ( india ) OR AFFILCOUNTRY ( india ) )	3,817,658
#5		( ( TITLE-ABS-KEY ( pregnan* ) OR TITLE-ABS-KEY ( mother* ) OR TITLE-ABS-KEY ( antenatal ) OR TITLE-ABS-KEY ( gestation* ) ) ) AND ( ( TITLE-ABS-KEY ( covid 19 vaccine* ) OR TITLE-ABS-KEY ( sars AND cov2 AND vaccine* ) OR TITLE-ABS-KEY ( pfizer AND biontech ) OR TITLE-ABS-KEY ( moderna ) OR TITLE-ABS-KEY ( vaxzevria ) OR TITLE-ABS-KEY ( astrazeneca ) OR TITLE-ABS-KEY ( sputnik ) OR TITLE-ABS-KEY ( sinovac ) OR TITLE-ABS-KEY ( covishield ) OR TITLE-ABS-KEY ( covaxin ) ) ) AND ( ( TITLE-ABS-KEY ( safety ) OR TITLE-ABS-KEY ( efficacy ) OR TITLE-ABS-KEY ( mortality ) OR TITLE-ABS-KEY ( hospitalization ) OR TITLE-ABS-KEY ( adverse AND event* ) OR TITLE-ABS-KEY ( aefi ) OR TITLE-ABS-KEY ( aefi AND adverse AND events AND following AND immunization ) OR TITLE-ABS-KEY ( effectiveness ) OR TITLE-ABS-KEY ( feto AND maternal AND outcome* ) OR TITLE-ABS-KEY ( pregnancy AND outcome* ) OR TITLE-ABS-KEY ( "Adverse neonatal outcome*" ) OR TITLE-ABS-KEY ( "perinatal death" ) OR TITLE-ABS-KEY ( "preterm birth" ) OR TITLE-ABS-KEY ( "small for gestational age" ) OR TITLE-ABS-KEY ( "low birth weight" ) OR TITLE-ABS-KEY ( "Apgar score" ) OR TITLE-ABS-KEY ( "neonatal hypoglycemia" ) OR TITLE-ABS-KEY ( "neonatal jaundice" ) OR TITLE-ABS-KEY ( "neonatal respiratory distress" ) OR TITLE-ABS-KEY ( "neonatal infection*" ) OR TITLE-ABS-KEY ( "congenital malformation*" ) OR TITLE-ABS-KEY ( "Neonatal Respiratory Distress Syndrome" ) OR TITLE-ABS-KEY ( "Congenital Abnormalit*" ) OR TITLE-ABS-KEY ( "perinatal death" ) OR TITLE-ABS-KEY ( "delivery outcome*" ) OR TITLE-ABS-KEY ( "Birth Outcome*" ) OR TITLE-ABS-KEY ( "gestational diabetes" ) OR TITLE-ABS-KEY ( preeclampsia ) OR TITLE-ABS-KEY ( "maternal infection*" ) OR TITLE-ABS-KEY ( "antepartum hemorrhage" ) OR TITLE-ABS-KEY ( "placental abruption" ) OR TITLE-ABS-KEY ( "premature rupture of membranes" ) OR TITLE-ABS-KEY ( prom ) OR TITLE-ABS-KEY ( "induction of labour" ) OR TITLE-ABS-KEY ( "mode of delivery" ) OR TITLE-ABS-KEY ( "postpartum hemorrhage" ) ) ) AND ( ( TITLE-ABS-KEY ( india ) OR AFFILCOUNTRY ( india ) ) )	148
Cochrane Library	#1	(pregnan*):ti,ab,kw OR (gestation):ti,ab,kw	96,992
	#2	MeSH descriptor: [Pregnant Women] explode all trees	1,055
	#3	MeSH descriptor: [Pregnancy] explode all trees	34,820
	#4	#1 OR #2 OR #3	97,324
	#5	(covid 19 vaccine*):ti,ab,kw OR (sars cov2 vaccine*):ti,ab,kw OR (pfizer biontech):ti,ab,kw OR (moderna):ti,ab,kw OR (Covishield):ti,ab,kw	3,143
	#6	(COVAXIN):ti,ab,kw OR (astrazeneca):ti,ab,kw OR (Sputnik):ti,ab,kw OR (Sinopharm):ti,ab,kw OR (Vaxzevria):ti,ab,kw	3,344
	#7	(Sinovac):ti,ab,kw OR (CoronaVac):ti,ab,kw	232
	#8	MeSH descriptor: [COVID-19 Vaccines] explode all trees	799
	#9	#5 OR #6 OR #7 OR #8	6,152
	#10	MeSH descriptor: [Safety] explode all trees	5,169
	#11	MeSH descriptor: [Vaccine Efficacy] explode all trees	106
	#12	MeSH descriptor: [Pregnancy Outcome] explode all trees	5,307
	#13	(safety):ti,ab,kw OR (mortality):ti,ab,kw OR (hospitalization):ti,ab,kw OR (adverse event*):ti,ab,kw OR (AEFI*):ti,ab,kw	528,821
	#14	(death*):ti,ab,kw OR (efficacy):ti,ab,kw OR (effectiveness):ti,ab,kw OR (fetomaternal outcome*):ti,ab,kw AND (feto maternal outcome*):ti,ab,kw	680,184
	#15	(pregnancy outcome*)	52,252
	#16	#10 OR #11 OR #12 OR #13 OR #14 OR #15	925,037
	#17	MeSH descriptor: [India] explode all trees	3,508
	#18	(india):ti,ab,kw	13,203
	#19	#17 OR #18	13,203
	#20	#4 AND #9 AND #16 AND #19	21

Screening and Data Extraction Process

Based on the keywords, articles were listed from the databases. The duplicates were reviewed and removed using NESTED Knowledge (https://knowledge.com/) [14]. Two independent reviewers (KD and AK) screened articles for eligibility sequentially. Initially, the title with abstract review was conducted, followed by full-text screening. Any discrepancies in the eligibility of the studies were fixed in discussion with a third reviewer (APG). The reasons for excluding the articles were documented. Data were extracted in MS Excel (Microsoft® Corp., Redmond, WA) using a standardized template. The key characteristics include study design, demography, intervention features (type and dose of vaccine), and outcomes (maternal, fetal, and pregnancy-related complications).

Risk of Bias (ROB) Assessment

Study quality was evaluated using design-specific tools such as the Newcastle-Ottawa Scale (NOS) [15] for cohort studies and the Joanna Briggs Institute (JBI) [16] for cross-sectional studies.

Statistical Analysis and Certainty in Evidence

A random effects model was used to calculate pooled estimates for outcomes (odds ratio) with maximum likelihood estimators. I² statistic assessed the heterogeneity among studies. Significant heterogeneity prompted the calculation of prediction intervals [17]. Subgroup analysis was planned to be undertaken if significant heterogeneity was present between the studies and an adequate number of studies were available. Publication bias assessment was planned but was not done as fewer studies qualified for the outcomes' meta-analyses. All analyses were done using RStudio software (Posit Software, Boston, MA) [18], with a significance threshold of p < 0.05. The certainty of pooled estimates of the outcomes was assessed and summarized using GRADEpro software (https://www.gradepro.org) according to the Grading of Recommendations Assessment, Development, and Evaluation (GRADE) methodology [19]. Single-arm studies without a comparator group were excluded from pooled estimates and were presented as a narrative synthesis.

Ethical Considerations

Ethics committee review and participant consent were not applicable to the study, since it was a systematic review. The protocol of the review has been registered with PROSPERO (CRD42024608801).

Results

**Figure 1:**
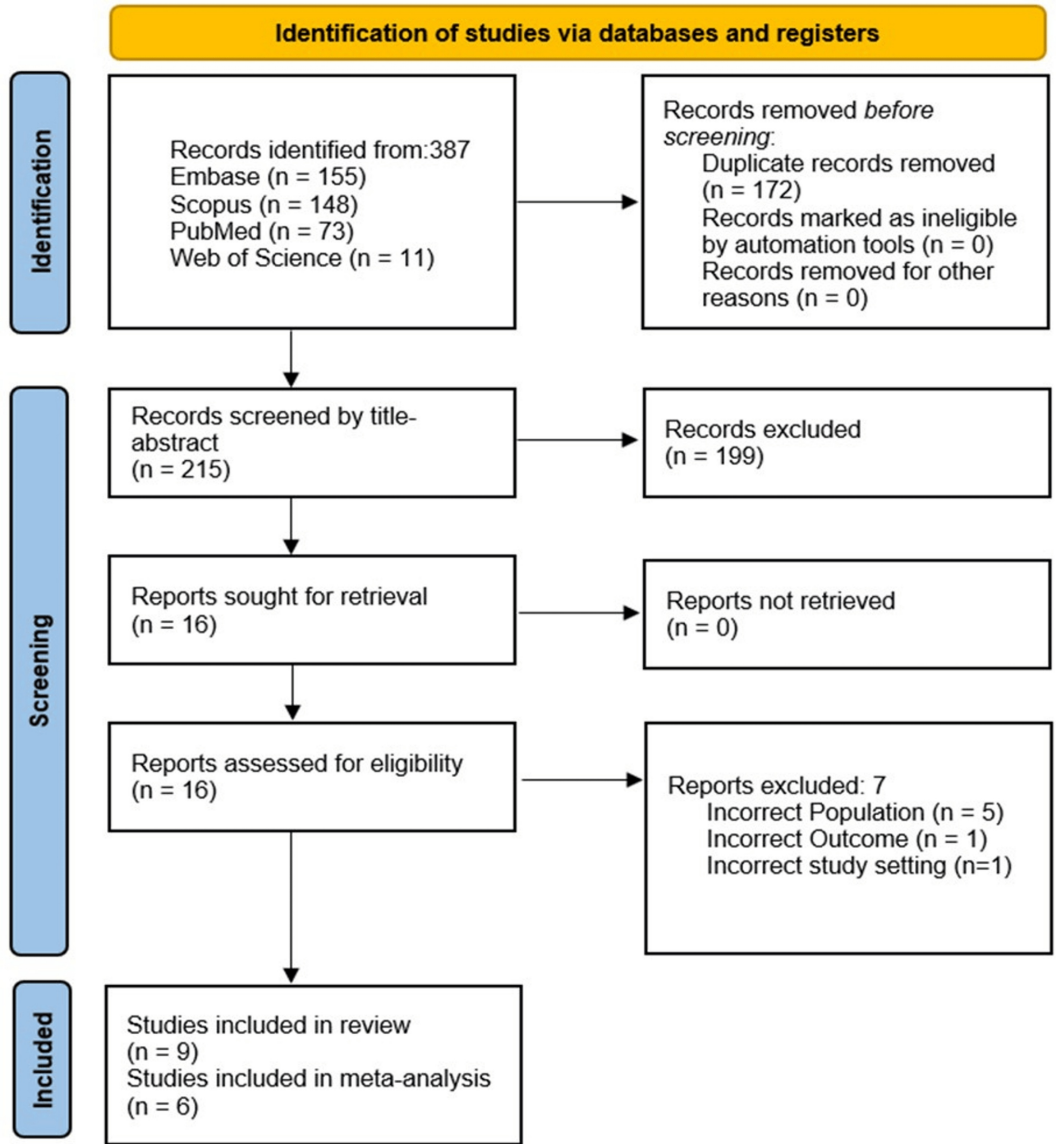
PRISMA flowchart showing the study selection process and studies included in the systematic review PRISMA: Preferred Reporting Items for Systematic Reviews and Meta-Analyses

The screening and selection process, using the PRISMA flowchart for SRMA, involved identifying 387 articles through a comprehensive search across databases (Figure 1). After deduplication, 172 duplicate entries were removed, leaving 215 unique articles for screening. Following title-abstract screening, 16 articles were shortlisted for full-text review. No additional articles were identified through bibliography screening or grey literature searches. During the full-text review, seven articles were excluded, resulting in a final selection of nine studies for inclusion in the SRMA.

The characteristics of the participants from individual studies are enumerated in Table 3. The pooled analysis included 5,630 pregnant women drawn from the selected studies. Of them, 3,030 had received ≥1 dose of a COVID-19 vaccine and were categorized as the exposed group (E), while 2,600 individuals were considered the control group (C), who were unvaccinated pregnant or vaccinated non-pregnant women. Sample sizes among studies varied, with the exposed group ranging from 50 to 1,170 participants and the control group from 100 to 1,443 participants. Five studies exclusively reported AEFI in the exposed group [10,20-23]. Two studies were undertaken on non-pregnant women with COVID-19 vaccination as the control group [24,25], while four studies were conducted between pregnant women who received COVID-19 vaccination and pregnant women who were not vaccinated [10,22,26,27]. Six studies have considered both Covishield and Covaxin in their exposed group [10,20-23,26], Covishield alone in two studies [24,27], and Covaxin alone in one study [25]. Except Pandey et al. [26], all other studies explicitly reported the number of pregnant women who received COVID-19 vaccines in the first, second, and third trimesters.

**Table 3:** Demographic details of study participants included in the systematic review *Vaccinated non-pregnant women ^$^Median age C: control group, E: exposed group, AEFI: adverse events following immunization, LSCS: cesarean section, SGA: small for gestational age, APGAR: appearance, pulse, grimace, activity, and respiration, NICU: neonatal intensive care unit, RDS: respiratory distress syndrome, GDM: gestational diabetes mellitus, LBW: low birth weight, PPH: postpartum hemorrhage, IUD: intrauterine death, MAS: meconium aspiration syndrome, PROM: premature rupture of membranes

Author and year	State	Study design	Vaccine	Number of patients (E/C)	Mean age (years)		Outcomes
					E	C	
Gandhi et al. (2022) [24]	Chandigarh	Prospective cohort study	Covishield	247/247*	25 (23-28)^$^	25 (22-28)^$^	AEFI: fever, body pain, weakness, injection site pain, swelling, cold, cough, sore throat
Gandhi et al. (2024) [27]	Chandigarh	Prospective cohort study	Covishield	280/129	25 (22-28)^$^	25 (22-28)^$^	Fetal outcomes: abortions, stillbirth, preterm delivery, postterm delivery, LSCS, low birth weight, SGA, APGAR, NICU admission, RDS, neonatal mortality; maternal outcomes: maternal anemia, GDM, preeclampsia, oligohydramnios
Goruntla et al. (2023) [20]	Andhra Pradesh	Prospective study	Covaxin, Covishield	420	21-35 (range)	21-35 (range)	AEFI: injection site pain, fever, myalgia, cough, cold, headache, fatigue, back pain, dizziness, vomiting
Kantharaja et al. (2023) [25]	Karnataka	Prospective observational study	Covaxin	100/100*	25.01	28.52	AEFI: fatigue, fever, injection site pain, myalgia, headache
Kolla et al. (2023) [21]	Andhra Pradesh	Prospective observational study	Covaxin, Covishield	50/-	21-35 (range)	-	AEFI: fever, headache, body ache; fetal outcomes: preterm delivery, LBW, NICU admission; maternal outcomes: LSCS, instrumental delivery
Pandey et al. (2023) [26]	Delhi	Retrospective cohort study	Covaxin, Covishield	105/295	27 (22-30)^$^	26 (22-29)^$^	Maternal outcomes: preeclampsia, PPH, LSCS; fetal outcomes: preterm delivery, IUD
Pandit et al. (2023) [22]	Gujarat	Retrospective cohort study	Covaxin, Covishield	531/1,443	-	-	AEFI: body ache, headache, fever, injection site pain, weakness; maternal outcomes: preterm delivery, postterm delivery; fetal outcomes: premature birth, LBW, congenital anomaly, NICU admission, IUD
Shree et al. (2024) [23]	Uttar Pradesh	Cross-sectional study	Covaxin, Covishield	1,170/-	20-35 (range)		AEFI: fever, headache, fatigue, muscle ache; maternal outcomes: instrumental delivery, LSCS, prolonged labor, maternal anemia, GDM, preeclampsia, hypothyroidism, PPH; fetal outcomes: preterm delivery, LBW, APGAR < 7, RDS, IUD, MAS, NICU admission
Tripathy et al. (2023) [10]	Odisha	Retrospective study	Covaxin, Covishield	127/154	28.24	28.12	AEFI: fever, headache, injection site pain, myalgia, vomiting; maternal outcomes: LSCS, maternal anemia, GDM, PROM, hypothyroidism, preeclampsia; fetal outcomes: preterm delivery, IUD, SGA, NICU admission

Three studies were single-arm studies without a comparator group [20,21,23]. Goruntla et al. reported AEFI incidence of 93.8%, with pain at injection site being the most common event (28.4%-29.6%) [20]. Kolla et al. from Andhra Pradesh reported mild symptoms following vaccination among 46% of the vaccinated pregnant women. Among the deliveries, 8% were preterm, and 16% of the neonates required neonatal intensive care unit (NICU) admission [21]. Shree et al. reported fever with chills and fatigue as the most common adverse events following the first and second doses of the COVID-19 vaccine. Overall, they reported no increase in the adverse fetomaternal outcomes following COVID-19 vaccination in pregnant women [23].

Outcomes of Pooled Studies

**Figure 2:**
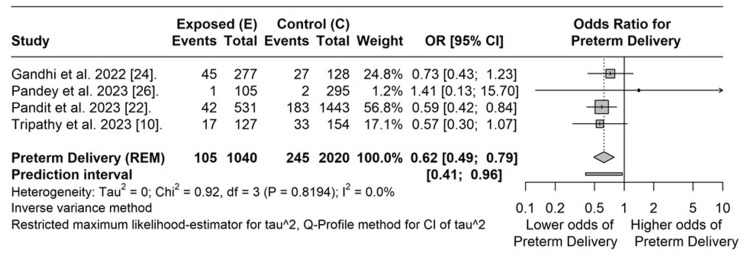
Pooled odds ratio for preterm delivery between vaccinated and unvaccinated pregnant women E: exposed group, C: control group, REM: random effects model, CI: confidence interval

Four cohort studies assessed the maternal outcome of preterm labor between mothers with COVID-19 vaccination and those without it [10,22,26,27]. The pooled odds ratio (OR) indicates a statistically significant lower odds of preterm labor in mothers with COVID-19 vaccination than in those mothers belonging to the control group (OR: 0.62, 95% CI: 0.49-0.79). The prediction interval was 0.41-0.96. No heterogeneity was observed between the studies (I² = 0%, p = 0.82) (Figure 2).

**Figure 3:**
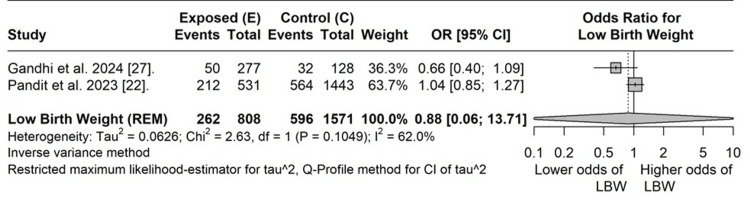
Pooled odds ratio for low birth weight between vaccinated and unvaccinated pregnant women LBW: low birth weight, E: exposed group, C: control group, REM: random effects model, CI: confidence interval

Two cohort studies reported the low birth weight between mothers with COVID-19 vaccination and those without [26,27]. The combined OR indicated a lower odds of low-birth-weight babies in mothers with COVID-19 vaccination than in those mothers belonging to the control group (OR: 0.88, 95% CI: 0.06-13.71). However, the findings were not statistically significant. Substantial heterogeneity was observed between the studies (I² = 62%, p = 0.10). Also, even individually, the studies did not report any significant association (Figure 3).

Pooled risk estimates for different outcomes between the exposed and unexposed groups are enumerated in Table 4.

**Table 4:** Pooled estimate for different outcomes between the exposed and unexposed groups AEFI: adverse events following immunization, OR: odds ratio, CI: confidence interval, NICU: neonatal intensive care unit

Outcome	Number of studies	Pooled estimate OR (95% CI)	I^2^	Prediction interval	p-value
AEFIs					
Any AEFI [24,25]	2	0.88 (0.00-268.11)	83%	-	0.02
Fever [24,25]	2	1.39 (0.20-9.57)	0%	-	0.38
Body ache [24,25]	2	1.67 (0.40-6.98)	0%	-	0.53
Injection pain [24,25]	2	0.72 (0.09-5.60)	0%	-	0.32
Weakness/fatigue [24,25]	2	0.90 (0.00-13046327.90)	79%	-	0.03
Fetomaternal outcomes					
Maternal anemia [10,27]	2	0.65 (0.02-26.51)	0%	-	0.43
Gestational diabetes mellitus [10,27]	2	0.53 (0.05-5.04)	0%	-	0.72
Preeclampsia [10,26,27]	3	1.13 (0.39-3.26)	0%	0.02-57.85	0.53
LSCS [10,26,27]	3	1.40 (0.76-2.58)	2%	0.22-8.99	0.36
Preterm delivery [10,22,26,27]	4	0.62 (0.49-0.79)	0%	0.41-0.96	0.82
Postterm delivery [26,27]	2	4.66 (0.00-39599993930.99)	94%	-	<0.01
Low birth weight [26,27]	2	0.88 (0.06-13.71)	62%	-	0.10
Congenital anomalies [26,27]	2	0.61 (0.01-35.31)	0%	-	0.59
NICU admission [10,22,27]	3	0.61 (0.08-4.55)	65%	0.00-28504.89	0.06
Intrauterine death [10,22,26]	3	0.85 (0.19-3.89)	0%	0.01-81.98	0.38
Premature birth [10,22]	2	0.59 (0.47-0.74)	0%	-	0.91
Small for gestational age [10,27]	2	0.35 (0.00-3361384.01)	63%	-	0.10

Other outcomes in AEFI (headache, injection site swelling, cough, cold, sore throat, dizziness, and vomiting) and fetomaternal outcomes (hypothyroidism, oligohydramnios, PPH, abortion, APGAR < 7, stillbirth, RDS, PROM, and neonatal mortality) did not have at least two studies to be qualified for meta-analysis. These outcomes are not reported in the meta-analysis; hence, they are reported in the qualitative synthesis (Table 5).

**Table 5:** Odds ratio for individual studies (qualitative synthesis) AEFI: adverse events following immunization, CI: confidence interval, APGAR: appearance, pulse, grimace, activity, and respiration

Outcome	Odds ratio (95% CI)
AEFI	
Headache [24]	3.05 (0.61-15.26)
Injection swelling [24]	1.47 (0.82-2.61)
Cough [24]	2.01 (0.18-22.29)
Cold [24]	1.00 (0.14 - 7.16)
Sore throat [24]	2.01 (0.18-22.29)
Dizziness [24]	0.50 (0.04-5.53)
Vomiting [24]	7.17 (0.88-58.76)
Fetomaternal outcomes	
Hypothyroidism [10]	1.25 (0.61-2.55)
Oligohydramnios [27]	1.39 (0.06-34.46)
Postpartum hemorrhage [26]	2.83 (0.18-45.60)
Abortion [27]	1.39 (0.14-13.46)
Stillbirth [27]	4.23 (0.23-79.13)
APGAR < 7 [10]	2.39 (1.10-5.20)
Respiratory distress syndrome [27]	0.46 (0.03-7.42)
Premature rupture of membranes [10]	1.73 (0.67-4.44)
Neonatal mortality [27]	1.39 (0.14-13.50)

Other AEFI outcomes, such as headache, injection site swelling, and sore throat, were similar between the groups (Table 5). Fetal outcomes such as stillbirth, RDS, and neonatal mortality were similar across the exposed group and the control group. The odds of an APGAR score of less than 7 (OR: 2.39, 95% CI: 1.10-5.20) were higher in the exposed group (Table 5).

Risk of Bias Assessment

ROB assessment is shown in Tables 6-8. Five cohort studies were graded as good quality. Two studies were assessed by the JBI prevalence tool, and two by the JBI analytical tool. All four studies had an overall appraisal of good and were hence included in the SRMA.

**Table 6:** Quality assessment of included cohort studies with the use of the Newcastle-Ottawa quality assessment tool *: one star rating, **: two-star rating, S1: representativeness of the exposed cohort, S2: selection of the non-exposed cohort, S3: ascertainment of exposure, S4: demonstration that outcome of interest was not present at the start of the study, C1: comparability of cohorts on the basis of the design or analysis, O1: assessment of outcome, O2: was follow-up long enough for outcomes to occur, O3: adequacy of follow-up of cohorts

Author and year	S1	S2	S3	S4	C1	O1	O2	O3	Overall quality
Gandhi et al. (2022) [24]	*	*	*	*	**	*	*	*	Good
Gandhi et al. (2024) [27]	*	*	*	*	**	*	*	*	Good
Pandey et al. (2023) [26]	*	*	*	*	**	*	*	*	Good
Pandit et al. (2023) [22]	*	*	*	*	*	*	*	*	Good
Tripathy et al. (2023) [10]	*	*	*	*	**	*	*	*	Good

**Table 7:** Quality assessment of included cross-sectional studies with the use of the JBI (analytical) quality assessment tool 1: Were the criteria for inclusion in the sample clearly defined? 2: Were the study subjects and the setting described in detail? 3: Was the exposure measured in a valid and reliable way? 4: Were objective, standard criteria used for the measurement of the condition? 5: Were confounding factors identified? 6: Were strategies to deal with confounding factors stated? 7: Were the outcomes measured in a valid and reliable way? 8: Was appropriate statistical analysis used? JBI: Joanna Briggs Institute

Author and year	1	2	3	4	5	6	7	8	Overall quality
Shree et al. (2024) [23]	Yes	Yes	Yes	Yes	No	Not applicable	Yes	Yes	Include
Kantharaja et al. (2023) [25]	Yes	Yes	Yes	Yes	No	Yes	Yes	Yes	Include

**Table 8:** Quality assessment of included cross-sectional study with the use of the JBI (prevalence) quality assessment tool 1: Was the sample frame appropriate to address the target population? 2: Were study participants sampled in an appropriate way? 3: Was the sample size adequate? 4: Were the study subjects and the setting described in detail? 5: Was the data analysis conducted with sufficient coverage of the identified sample? 6: Were valid methods used for the identification of the condition? 7: Was the condition measured in a standard, reliable way for all participants? 8: Was there appropriate statistical analysis? 9: Was the response rate adequate, and if not, was the low response rate managed appropriately? JBI: Joanna Briggs Institute

Author and year	1	2	3	4	5	6	7	8	9	Overall quality
Goruntla et al. (2023) [20]	Yes	Unclear	Yes	Yes	Unclear	Yes	Yes	Yes	Unclear	Include
Kolla et al. (2023) [21]	Yes	Unclear	No	Yes	Unclear	Yes	Yes	Yes	Unclear	Include

Certainty of Evidence: GRADE Profile

Very low certainty of evidence for AEFIs and APGAR < 7 for the COVID-19 vaccination was found. Low birth weight also showed a very low certainty in evidence due to serious imprecision concerns, primarily owing to the wide confidence interval. A low certainty of evidence for preterm delivery was noted. Although none of the GRADE domains had serious issues for preterm delivery, the certainty of evidence could not be rated as moderate or high, owing to a lack of large effect size and study design (Tables 9, 10).

**Table 9:** GRADE profile for the overall AEFIs following COVID-19 vaccination (pregnant versus non-pregnant women) Explanations: a. The effect estimates of the two included studies are in different directions. b. The confidence interval is very wide (0.00-268.11), and the optimal information size has not been met (optimal information size taken >2,000 for dichotomous outcomes). GRADE: Grading of Recommendations Assessment, Development, and Evaluation, CI: confidence interval, OR: odds ratio, COVID-19: coronavirus disease 2019, AEFI: adverse events following immunization

Certainty assessment							Summary of findings				
Participants (studies)	Risk of bias	Inconsistency	Indirectness	Imprecision	Publication bias	Overall certainty of evidence	Study event rates (%)		Relative effect (95% CI)	Anticipated absolute effects	
							Pregnant	Non-pregnant		Risk with non-pregnant women	Risk difference with COVID-19 vaccine (pregnancy)
AEFIs											
694 (2 non-randomized studies) [24,25]	Not serious	Serious^a^	Not serious	Extremely serious^b^	None	⨁◯◯◯ Very low^a,b^	213/347 (61.4%)	214/347 (61.7%)	OR: 0.88 (0.00-268.11)	213/347 (61.4%)	31 fewer per 1,000 (from -- to 384 more)

**Table 10:** GRADE profile for the fetomaternal outcomes following COVID-19 vaccination in pregnant women (vaccinated versus unvaccinated pregnant women) Explanations: a. The confidence Interval is very wide (0.06-13.71). b. The optimal information size has not been met (optimal information size taken >2,000 for dichotomous outcomes). GRADE: Grading of Recommendations Assessment, Development, and Evaluation, CI: confidence interval, OR: odds ratio, COVID-19: coronavirus disease 2019, APGAR: appearance, pulse, grimace, activity, and respiration

Certainty assessment							Summary of findings				
Participants (studies)	Risk of bias	Inconsistency	Indirectness	Imprecision	Publication bias	Overall certainty of evidence	Study event rates (%)		Relative effect (95% CI)	Anticipated absolute effects	
							Vaccinated	Unvaccinated		Risk with unvaccinated pregnant women	Risk difference with COVID-19 vaccine (pregnancy)
Low birth weight											
2,379 (2 non-randomized studies) [26,27]	Not serious	Not serious	Not serious	Serious^a^	None	⨁◯◯◯ Very low^c^	596/1571 (37.9%)	262/808 (32.4%)	OR: 0.88 (0.06-13.71)	596/1571 (37.9%)	30 fewer per 1,000 (from 344 fewer to 514 more)
Preterm delivery											
3,060 (4 non-randomized studies) [10,22,26,27]	Not serious	Not serious	Not serious	Not serious	None	⨁⨁◯◯ Low	245/2020 (12.1%)	105/1040 (10.1%)	OR: 0.62 (0.62-0.79)	245/2020 (12.1%)	42 fewer per 1,000 (from 42 fewer to 23 fewer)
APGAR < 7											
274 (1 non-randomized study) [10]	Not serious	Not serious	Not serious	Serious^b^	None	⨁◯◯◯ Very low^d^	11/149 (7.4%)	20/125 (16.0%)	OR: 2.39 (1.10-5.20)	11/149 (7.4%)	86 more per 1,000 (from 7 more to 219 more)

Discussion

Historically, vaccination during pregnancy has been recognized as a pivotal public health intervention, offering primary prevention against communicable diseases. Evidence demonstrates its substantial role in lowering adverse maternal and perinatal outcomes, highlighting its enduring impact on improving health outcomes across populations [28]. The current SRMA is the first of its kind to ascertain the safety of COVID-19 vaccines among pregnant mothers in India. The majority of the included studies were from the northern states of India.

Vaccination against COVID-19 offers dual benefits, both to the mother and the neonate; however, the mothers are afraid of adverse outcomes and side effects of vaccination [29]. This meta-analysis shows that the mothers who had received COVID-19 vaccines during pregnancy had a significantly lower risk for preterm delivery in comparison with the group that was not vaccinated, albeit with insufficient strength of evidence. This finding broadly reverberates the work from other meta-analyses conducted in the global context, especially of high-income countries [30,31]. It is encouraging to observe similar findings in the living systematic review conducted by Ciapponi et al., pooling data from 177 studies globally [32]. Reduced risk of preterm delivery in vaccinated mothers was also supported by various other meta-analyses; however, the finding was statistically insignificant [33-36].

A strong association between COVID-19 infection and low birth weight in newborns has been reported in the literature [37,38]. Prior studies conducted in the global context have addressed this important outcome of pregnancy in vaccinated pregnant mothers, in which they found no relationship between COVID-19 vaccination status and birth weight in newborns [33,35,39]. Consistent with the literature, this meta-analysis reported a reduced risk of low birth weight in COVID-19 vaccinated mothers; however, the finding is statistically insignificant, and the included studies are heterogeneous in nature. Small-for-gestational babies were not associated with COVID-19 vaccination in the index SRMA, which seems consistent with the earlier studies [30,32,33]. The low birth weight may not be primarily due to infection with COVID-19 since low birth weight is multifactorial, such as health system-related factors and maternal well-being, among others [37].

Pooled analysis comparing the COVID-19 vaccination status in pregnant women and the risk of anemia showed no association in this SRMA. However, anemia has been reported as a rare phenomenon after COVID-19 vaccines in the general population [40]. A possible explanation for this might be the autoimmune cross-reactivity with the red blood cell antigens [40]. COVID-19 vaccination has no association with gestational diabetes mellitus (GDM) among pregnant women in India. These results are in agreement with those obtained by Dick et al. [41], while they differ from the observations of Marchand et al. [31], where they found a higher chance of occurrence of GDM with COVID-19 vaccination. However, with a lack of data regarding the timing of the diagnosis of diabetes in most of the studies, this finding must be interpreted with caution. The results of this review found no association between hypertensive disorders of pregnancy and COVID-19 vaccines. This resonates with the systematic review done by Ciapponi et al., which pooled data from 177 studies globally [32]. These findings were supported by Ma et al. [35], Tormen et al. [34], and Pratama et al. [39] as well.

With respect to mode of delivery, the current meta-analysis found no association between COVID-19 vaccination and cesarean section rates in pregnant mothers. Ding et al. found that the vaccinated mothers had a significantly reduced risk for cesarean section after addressing the potential confounders [33]. These results match those observed in earlier studies [30,35,39]. In contrast, Marchand et al., which pooled studies mainly from the USA and Israel, reported a higher risk of cesarean section in vaccinated pregnant women [31]. This contradictory finding can be attributed to differing context, maternal request for cesarean section [42], and other comorbid health conditions of pregnancy [43].

Adverse fetal and neonatal outcomes induced an immense sense of fear of vaccination uptake during COVID-19. No significant association was found between COVID-19 vaccination and congenital anomalies in the studies reviewed. These findings are supported by the SRMAs conducted by Tormen et al. [34], Ciapponi et al. [32], and Marchand et al. [31], which were done in a global context. Ding et al., in their pooled analysis, found that vaccination in the initial trimester had no relationship with congenital anomalies [33]. This shows the scope for future research exploring the outcomes with data on the timing of vaccination in pregnancy.

COVID-19 vaccines were reported to offer reduced risk of NICU admission [33]. However, in most other studies, they observed no significant difference in vaccination status and NICU admissions [30-32,34,35,39]. Some studies reported a reduced risk of NICU admission, but the majority, including ours, found no significant association. Thus, overall, studies in pregnant mothers exploring the impact of COVID-19 infection on NICU admissions showed inconsistent results. Fetal loss in pregnancy occurs due to multiple causes: birth defects, chromosomal anomalies, fetal infection, and placental abruption [44]. In this SRMA, intrauterine deaths were not associated with COVID-19 vaccination. There are similarities between findings in this study and those described by Shafiee et al. [30], Ma et al. [35], and Pratama et al. [39]. However, these studies had measured stillbirth as the outcome. Ding et al. reported a significantly reduced risk of stillbirth in COVID-19 vaccinated mothers [33]. This finding is contrary to that of Tormen et al., which reported increased risk of pregnancy loss, but the finding was insignificant [34].

The risk of AEFIs with COVID-19 vaccination in pregnancy is similar to non-pregnant vaccinated women, albeit from only two studies in the current review. The AEFI rates analyzed in the current SRMA, such as injection site pain, fever, body ache, and weakness, were similar to those reported by earlier studies [39,45], and serious AEFI events were rare. Several reports have shown that adverse effects occur more frequently with repetitive dosages [45]. This has reverberated in one of the studies from the current review, where AEFI rates were 72.07%-76.13% after two doses, while they were between 18.52% and 27.93% following the first dose [20]. However, Kantharaja et al. reported a similar rate of AEFIs following the first dose (35.9%) and the second dose (43.5%) of the vaccine in pregnant women [25]. Also, the timing of vaccination (trimester) did not show a significant effect on the fetomaternal outcomes in the current review. The evidence synthesis shows that outcomes such as APGAR score < 7 in five minutes, abortion, and postpartum hemorrhage were not associated with the COVID-19 vaccination.

Studies reporting on the efficacy and effectiveness could not be found in Indian settings. A global-level meta-analysis revealed that COVID-19 vaccination is significantly effective in reducing SARS-CoV-2 infections and related hospitalizations [34].

Strengths and Limitations

This is the first meta-analysis in the Indian context, exploring the safety of vaccination for COVID-19 in pregnant mothers by exploring major databases. Nevertheless, certain limitations are present in the review. First, all the studies included were observational study designs. Second, there were a low number of studies, and for some outcomes, there was high heterogeneity among studies. Third, most of the studies included were from the northern part of India, depicting less representation from northeastern and southern states, and only two vaccines, Covaxin and Covishield, were considered; as a result, the broader applicability of the findings is limited. Fourth, adequate information on breakthrough COVID-19 infection, antibody titers, the timing of vaccine administration, and booster doses was not available in the included studies. Thus, efficacy and effectiveness could not be assessed in the current review.

## Conclusions

Overall, in India, vaccination against COVID-19 may not be associated with AEFIs, adverse pregnancy, and fetal and neonatal outcomes, with a protective effect against preterm. However, the conclusions are limited by low certainty of evidence and generalizability. Additionally, no studies could be found on the efficacy and effectiveness of the vaccine. Notwithstanding these limitations, from a safety perspective, the review can enhance the confidence among pregnant mothers for COVID-19 vaccination in the most populous country in the world. This can assist efforts in addressing hesitancy, which in turn will improve vaccine coverage among pregnant women in India. Further research studies exploring efficacy, effectiveness, other regions, vaccine types, and administration timings in different trimesters and booster doses are required to enhance the evidence in this area in the Indian context.

## Appendices

Table 11 presents the PRISMA checklist adapted for the current study. 

**Table 11:** PRISMA checklist (2020) PRISMA: Preferred Reporting Items for Systematic Reviews and Meta-Analyses

Section and topic	Item #	Checklist item	Location where the item is reported
Title			
Title	1	Identify the report as a systematic review.	Page 1
Abstract			
Abstract	2	Made as per the journal guidelines	Page 1
Introduction			
Rationale	3	Describe the rationale for the review in the context of existing knowledge.	Pages 2,3
Objectives	4	Provide an explicit statement of the objective(s) or question(s) the review addresses.	Page 3
Methods			
Eligibility criteria	5	Specify the inclusion and exclusion criteria for the review and how studies were grouped for the syntheses.	Supplementary Table S2
Information sources	6	Specify all databases, registers, websites, organizations, reference lists, and other sources searched or consulted to identify studies. Specify the date when each source was last searched or consulted.	Page 4
Search strategy	7	Present the full search strategies for all databases, registers, and websites, including any filters and limits used.	Supplementary Table S3
Selection process	8	Specify the methods used to decide whether a study met the inclusion criteria of the review, including how many reviewers screened each record and each report retrieved, whether they worked independently, and if applicable, details of automation tools used in the process.	Page 4
Data collection process	9	Specify the methods used to collect data from reports, including how many reviewers collected data from each report, whether they worked independently, any processes for obtaining or confirming data from study investigators, and if applicable, details of automation tools used in the process.	Page 4
Data items	10a	List and define all outcomes for which data were sought. Specify whether all results that were compatible with each outcome domain in each study were sought (e.g., for all measures, time points, and analyses), and if not, the methods used to decide which results to collect.	Page 4
	10b	List and define all other variables for which data were sought (e.g., participant and intervention characteristics, and funding sources). Describe any assumptions made about any missing or unclear information.	Page 4
Study risk of bias assessment	11	Specify the methods used to assess risk of bias in the included studies, including details of the tool(s) used, how many reviewers assessed each study and whether they worked independently, and if applicable, details of automation tools used in the process.	Page 4
Effect measures	12	Specify for each outcome the effect measure(s) (e.g., risk ratio and mean difference) used in the synthesis or presentation of results.	Page 4
Synthesis methods	13a	Describe the processes used to decide which studies were eligible for each synthesis (e.g., tabulating the study intervention characteristics and comparing against the planned groups for each synthesis (item #5)).	Pages 4,5
	13b	Describe any methods required to prepare the data for presentation or synthesis, such as handling of missing summary statistics, or data conversions.	NA
	13c	Describe any methods used to tabulate or visually display the results of individual studies and syntheses.	Pages 4,5
	13d	Describe any methods used to synthesize results and provide a rationale for the choice(s). If meta-analysis was performed, describe the model(s), method(s) to identify the presence and extent of statistical heterogeneity, and software package(s) used.	Pages 4,5
	13e	Describe any methods used to explore possible causes of heterogeneity among study results (e.g., subgroup analysis and meta-regression).	NA
	13f	Describe any sensitivity analyses conducted to assess the robustness of the synthesized results.	NA
Reporting bias assessment	14	Describe any methods used to assess the risk of bias due to missing results in a synthesis (arising from reporting biases).	Page 4
Certainty assessment	15	Describe any methods used to assess certainty (or confidence) in the body of evidence for an outcome.	Page 5
Results			
Study selection	16a	Describe the results of the search and selection process, from the number of records identified in the search to the number of studies included in the review, ideally using a flow diagram.	Figure 1
	16b	Cite studies that might appear to meet the inclusion criteria, but which were excluded, and explain why they were excluded.	Page 5
Study characteristics	17	Cite each included study and present its characteristics.	Page 5, Table 1
Risk of bias in studies	18	Present assessments of risk of bias for each included study.	Page 6, Table S4-S6
Results of individual studies	19	For all outcomes, present, for each study: (a) summary statistics for each group (where appropriate) and (b) an effect estimate and its precision (e.g., confidence/credible interval), ideally using structured tables or plots.	Figure 2,3, Table S7
Results of syntheses	20a	For each synthesis, briefly summarize the characteristics and risk of bias among contributing studies.	Page 6, Table S4-S6
	20b	Present the results of all statistical syntheses conducted. If meta-analysis was done, present for each the summary estimate and its precision (e.g., confidence/credible interval) and measures of statistical heterogeneity. If comparing groups, describe the direction of the effect.	Figures 2,3, Table S7
	20c	Present the results of all investigations of possible causes of heterogeneity among study results.	NA
	20d	Present results of all sensitivity analyses conducted to assess the robustness of the synthesized results.	NA
Reporting biases	21	Present assessments of risk of bias due to missing results (arising from reporting biases) for each synthesis assessed.	NA
Certainty of evidence	22	Present assessments of certainty (or confidence) in the body of evidence for each outcome assessed.	Page 7
Discussion			
Discussion	23a	Provide a general interpretation of the results in the context of other evidence.	Pages 7-9
	23b	Discuss any limitations of the evidence included in the review.	Page 10
	23c	Discuss any limitations of the review processes used.	Page 10
	23d	Discuss implications of the results for practice, policy, and future research.	Page 10
Other information			
Registration and protocol	24a	Provide registration information for the review, including register name and registration number, or state that the review was not registered.	Page 1
	24b	Indicate where the review protocol can be accessed, or state that a protocol was not prepared.	Page 1
	24c	Describe and explain any amendments to information provided at registration or in the protocol.	NA
Support	25	Describe sources of financial or non-financial support for the review, and the role of the funders or sponsors in the review.	Title page
Competing interests	26	Declare any competing interests of review authors.	Title page
Availability of data, code, and other materials	27	Report which of the following are publicly available and where they can be found: template data collection forms; data extracted from included studies; data used for all analyses; analytic code; any other materials used in the review.	Title page
